# Long-Term Effect of Different Physical Activity Levels on Subclinical Atherosclerosis in Middle-Aged Men: A 25-Year Prospective Study

**DOI:** 10.1371/journal.pone.0085209

**Published:** 2014-01-20

**Authors:** Magdalena Kwaśniewska, Anna Jegier, Tomasz Kostka, Elżbieta Dziankowska-Zaborszczyk, Ewa Rębowska, Joanna Kozińska, Wojciech Drygas

**Affiliations:** 1 Department of Social and Preventive Medicine, Medical University of Lodz, Lodz, Poland; 2 Department of Sports Medicine, Medical University of Lodz, Lodz, Poland; 3 Department of Geriatrics, Medical University of Lodz, Lodz, Poland; 4 Department of Cardiovascular Epidemiology and Prevention, Institute of Cardiology, Warsaw, Poland; Wageningen University, Netherlands

## Abstract

**Background:**

The purpose of the study was to investigate the influence of lifetime physical activity (PA) on selected indices of atherosclerosis in longitudinal observation of middle-aged men.

**Methods:**

The subject of the study was a cohort of 101 men (mean age 59,7±9,0 years), free of cardiovascular symptoms and treatment, participating in follow-up examinations in the years 1985/90-2011/12. Self-report PA was assessed by interviewer-administered Seven-Day PA Recall and Historical PA questionnaire. Subclinical atherosclerosis was measured by assessing the coronary artery calcification (CAC) according to Agatston's method using multi-slice computed tomography; the carotid intima-media thickness (IMT) using high-resolution B-mode ultrasound; and the reactive hyperemia index (RHI) using peripheral arterial tonometry (EndoPAT2000). The participants were initially divided into three groups according to tertiles of exercise-related energy expenditure (EE) in kcal/week at baseline, i.e. <2050 (low-to-moderate; n = 33), 2050–3840 (high; n = 34), >3840 (very high; n = 34).

**Results:**

The low-to-moderate, high and very high PA groups were comparable in terms of age and atherosclerosis risk factors at baseline. No linear relationship was found between PA and CAC, IMT and RHI. Men who maintained low-to-moderate (n = 26), high (n = 21) and very high (n = 15) PA level had the mean CAC of 286.1±361.9, 10.7±28.9, and 106.1±278.3 (p<0.001 for low-to moderate vs high; p<0.05 for low-to-moderate vs very high); the mean IMT of 0.751±0.19 mm, 0,641±0.26 mm, and 0.750±0.60 mm (p>0.05); and the mean RHI of 1.69±0.4, 2.00±0.4, and 2.13±0.5 (p for trend = 0.050), respectively. No cases of CAC>400, IMT ≥0.9 and RHI<1.67 were noted only among men with maintained high PA level. At final examination men with high and very high PA had more favorable cardiometabolic profile than men with lower PA.

**Conclusions:**

Maintaining regular high PA level through young and middle adulthood may protect against atherosclerosis as measured by CAC, IMT and RHI.

## Introduction

There is convincing body of evidence on the independent role of physical activity (PA) in primary prevention of cardiovascular diseases (CVD) [1]–[4]. Reduced CVD morbidity and mortality in physically active individuals has been repeatedly shown to be associated with beneficial effects on major risk factors including lipid disorders, hypertension, diabetes or obesity [5], [6]. However, data on the relationship between PA and subclinical atherosclerosis are scarce. Usually used measures of the presence and extent of subclinical atherosclerosis are coronary artery calcification (CAC) and carotid intima-media thickness (IMT)[7], [8]. Few prior studies that have examined this topic provided inconsistent results. Some authors found an inverse association between CAC or IMT and PA [9], [10] while others have reported weak or not significant relationships [11]–[13]. It is probable that observed heterogeneity of the obtained findings result from different methodology including various socioeconomic groups of participants, different domains of PA measured as well as PA assessment techniques. Most of the previous studies have adopted a cross-sectional design, so they could not infer the cause and effect relation. Moreover, substantial percent of the subjects participating in prior studies were taking medications known for their antiatherogenic properties.

Therefore, we decided to examine the relationship between PA and selected indices of subclinical atherosclerosis in long-term observation of a healthy socioeconomically homogeneous cohort of middle-aged men. In order to reduce a caveat concerning antiatherogenic effects of some medications, the study group consisted of persons who were not taking any agents modifying CVD risk. Apart from assessing CAC and IMT, we also evaluated peripheral microvascular endothelial function due to the meaning of endothelial dysfunction as the earliest detectable disturbance in the natural history of atherosclerosis [14].

## Materials and Methods

All the subjects were provided with a written information about the purpose and methodology of the study. The protocol of the project has been approved by the Medical University of Lodz Ethics Committee, and the written informed consent was obtained from all the participants. All clinical investigation have been conducted according to the principles expressed in the Declaration of Helsinki.

### Subjects

As it was described in our previous reports, the initial database of the Healthy Men Clinic and the Department of Preventive Medicine, Medical University of Lodz (Poland) was composed of 856 volunteers examined in the years 1975-80 [15]. However, more detailed anthropometric and laboratory measurements (including waist circumference, triglycerides and uric acid) have been collected since 1985. Therefore, the initial database created for the purpose of the present project consists of data gathered in the years 1985-90.

Subjects were eligible for this study if they participated in the full panel of diagnostic procedures during at least five examinations between baseline in 1985/90 and final in 2011/12. Subjects were further considered to be eligible if they were asymptomatic and free from chronic diseases, any important disability or dementia. As some medications are known to affect the process of atherosclerosis, we also excluded individuals taking drugs modifying CVD risk (including aspirin, lipid-lowering, glucose-lowering and blood pressure-lowering agents).

Of the initial 577 subjects who participated in baseline structured health check-up in 1985/90, a total of 368 men participated in a follow-up in 2003/5 realized within a scientific grant of the Ministry of Science and Higher Education. Data gathered in 2003/5 were assessed for eligibility and 193 subjects met preliminary inclusion criteria. Of the 193 subjects personally invited by mail, 10 men did not respond, 3 died in the years 2003–2012 (1 stroke, 1 cancer, 1 car accident), 23 were diagnosed with chronic diseases and 24 was taking CVD drugs (information collected during a phone call). Of the 134 men (mean age 62,1±3.6 yrs) who attended the Clinic in 2011/12, we disqualified 33 subjects due to taking agents modifying CVD risk and/or abnormalities found during physical examination, echocardiography or exercise test.

Therefore, the final cohort consisted of 101 men aged 50–77 years (mean age 59.7±9.0 yrs; mean observation period: 24.7±4.1 yrs) who regularly attended our Department in the years 1985–2012. Most of them (89%) participated in more than five examinations (mean 5.8±2.7). The subjects were white men, predominantly married, white collar workers with university or secondary educational level whose occupational activity was low. Most participants with at least moderate PA level (>1000 kcal/wk) had been involved in non-competitive sports activities of endurance type, such as running, bicycling, swimming, or basketball for several years.

Persons who were disqualified from this analysis (due to CVD disease or treatment) had substantially different CVD risk in comparison to the final study cohort throughout the whole observation. According to baseline data (1985/90) the disqualified group differed significantly from the studied group in terms of the prevalence of smoking (51.8% vs 17.8%, p<0.001), mean BMI (26.9 vs 24.8 kg/m^2^, p<0.01), systolic blood pressure (127.4 vs 118.9 mmHg, p<0.001), total cholesterol (5.09 vs 4.84 mmol/l, p<0.001). According to data gathered during subsequent follow-ups the differences remained significant and in 2003/5 the prevalence of hypertension, hypercholesterolemia, obesity, ischaemic heart disease was 43.2%, 79.2%, 21.0% and 11.9% among persons excluded from the analysis.

### Protocol and measures

In the years 1985–2005 all subjects participated in a similar panel of procedures including a detailed interviewer-administered questionnaire, anthropometric and biochemical measurements, resting electrocardiogram and the graded submaximal exercise test. The 2011/2012 follow-up was divided into two stages. The procedures of the first stage consisted of blood sample collection, interviewer-administered questionnaire, standard physical examination, anthropometric measurements, resting electrocardiogram, echocardiography and the graded submaximal exercise test. The subjects who met all inclusion criteria were qualified to the second stage of the projects, i.e. multi-slice computed tomography (MSCT) of the coronary arteries, ultrasound scanning of the carotid arteries, and peripheral artery tonometry (PAT).

MSCT and USG were preformed in the Military Teaching Hospital (Lodz, Poland), while all other examinations took place in the Department of Preventive Medicine of the Medical University of Lodz.

The subjects were asked to report to the center between 8:00 and 9:00 a.m. after overnight fasting for a minimum of 12 h, after overnight rest, refraining from physical exercises, smoking and alcohol for at least 12 h before laboratory measurements. After fasting blood drawing, all the participants were given a light meal, and a multidimensional assessment was performed on each subject. During the interview all the participants provided data on demographic and socio-economic status, smoking, dietary pattern, physical activity level, medical history, family history and quality of life.

### Physical activity assessment

In the years 1985–2002 data on physical activity was collected during the medical interview. The level of habitual leisure-time PA (LTPA), including commuting PA, during the previous year was estimated. Exercise-related energy expenditure (EE) was calculated on the basis of the amount of hours earmarked for weekly recreational sport activities (kcal/week) according to the tables of Fox et al. [16]. Since 2003 PA was also assessed by the Seven-Day PA Recall Questionnaire and by the historical PA according to Kriska and Caspersen [17]. As PA level may vary within individuals across time, specific categories were created to reflect long-term PA patterns. Based on the baseline LTPA, the participants were divided into three groups according to tertiles of EE: <2050 kcal/week, 2050–3840 kcal/week, and >3840 kcal/week and defined as low-to moderate, high and very high PA level, respectively. Next, we analyzed PA level throughout the whole observation. Based on the mean EE gathered at two-thirds of all follow-up examinations we defined subgroups of maintained, increased and decreased PA level.

Historical PA was estimated for the following periods of life: 12–34, 35–49, over 50 years old period, for the last 5 and 10 years and for the whole period from 12^th^ year of life to the day of examination. All PAs were summed up according to hours per week, weeks of activity during the month, months of activity during the year, and years of activity during a period. The estimated number of hours during a period was divided by the number of years. As a result, all the measures of historical PA are expressed as hours per year for a given period.

In order to assess aerobic fitness the graded submaximal exercise test was carried out on a Monark type 818E (Stockholm, Sweden) bicycle ergometer with 30 W increments every 3 min to achieve at least 85% of maximal age-predicted HR (220-age). Maximal oxygen consumption (VO_2max_), as a measure of aerobic fitness, was calculated by indirect method [18].

### Biochemical and anthropometric measurements

Fasting blood samples were drawn from the antecubital vein. Enzymatic methods were used to determine serum total cholesterol, glucose, triglycerides, uric acid concentrations (COBAS INTEGRA 400 Plus, Roche). HDL cholesterol (HDL-C) was measured by the precipitation method. LDL cholesterol (LDL-C) was estimated using the Friedewald formula. Anthropometric data were collected by standard methods. Body weight was measured to the nearest 100 g on calibrated scales (in light indoor clothes and without shoes). Height was measured with a stadiometer (without shoes) to the nearest 0.5 cm. Waist circumference was measured with a tape measure at the middle of the distance between the lowest rib and the iliac crest (in underwear, standing position) to the nearest 0.5 cm. Body mass index (BMI) was calculated as weight (kilograms) divided by square of height (meters). Skinfold measurements were taken at four sites: triceps, biceps, below the spatula, and above the ileum. The percentage of body fat was estimated according to Durnin and Womersley [19].

### Markers of subclinical atherosclerosis

#### Coronary artery calcification

CAC scores were evaluated using the 64-slice computed tomography scanner (SOMATOM Sensation 64, Siemens Medical Solutions, Forchheim, Germany) and with Syngo CaScore automatic analysis software (Siemens Healthcare, Forchheim, Germany). All scans were performed in cranio-caudal direction during inspiratory breathold with prospective electrocardiogram ECG-triggering (gantry rotation time, 330 ms; temporal resolution, 83–165 ms). CAC scores were separately obtained for each of the main epicardial coronary arteries (left main artery, left anterior descending artery, left circumflex artery, and right coronary artery and summed to obtain total CAC. The total CAC score was generated as per the Agatston method and reported in Agatston units (AU) [20]. As no substantial differences in PA level and CVD risk were found between the groups with CAC score of 1–10 and 11–100 as well as 101–400 and >400 AU, the comparisons were performed between the three categories of CAC: 0, 1–100, and >100 AU.

#### Intima-media thickness

Measurement of arterial wall thickness as a validated surrogate marker for atherosclerosis was performed and the presence of carotid atherosclerosis was defined as increased carotid IMT (>0.9 mm) [21], [22]. Carotid IMT was measured using ultrasound scanning. Images of the carotid arteries were acquired using the Siemens Acuson (Mountainview, CA) S2000 ultrasound system equipped with a 9.0–4.0 MHz transducer. Images of the right and left common carotid and internal carotid arteries were captured, including the investigation of the near and far walls. Mean IMT was defined as the mean of the IMT of the proximal and distal walls for both sides of the CCA at the available 5 points, i.e. 10, 15, 20, 25 and 30 mm proximal to carotid bulb. A single operator blinded to other analyzed variables of the participants performed all scans and offline analyses.

#### Reactive hyperemia

Peripheral arterial tonometry signals were obtained using the EndoPAT 2000 device (Itamar Medical Inc., Caesarea, Israel) in participants resting in the supine position in a quiet, temperature-controlled environment set at about 22°C after an overnight fast. Subjects were also instructed to refrain from smoking and strenuous exercise at least 12 hrs before the examination. Full details of the probe technology and the basis of measurements have been previously described [23]–[25]. Briefly, a PAT finger probe was placed on each index finger. Pulsatile volume changes of the distal digit induced pressure alterations in the finger cuff, which were sensed by pressure transducer and transmitted to and recorded by the EndoPAT 2000 device. Blood pressure (BP) and heart rate (HR) were measured by an automated BP monitor. Endothelial function was assessed via RH-PAT index. The ratio of the PAT signal after cuff release compared with baseline was calculated through a computer algorithm automatically normalizing for baseline signal and indexed to the contralateral arm. The estimated ratio reflects the RHI. RHI values <1.67 were considered abnormal.

#### Statistical analysis

Continuous variables are expressed as mean ± standard deviation (SD) or median (if not standard distribution). Fisher exact test, the Kruskall-Wallis test, Dunnett's test and chi-square test as well as chi-square test with Yate's correction were used in order to assess distribution of CAC, IMT and RHI according to the level of PA to compare differences between groups. Spearman's correlation was used to evaluate the association between CAC, RHI and IMT, and other continuous variables. The correlations were adjusted to WC, BMI, BP, lipids, glucose, uric acid and VO_2_max. Mann-Whitney test was used to compare historical LTPA during different life periods. A p value <0.05 was considered statistically significant. All statistical analyses were performed with STATISTICA Windows XP version 9.1.

## Results

Table 1 presents distribution of demographic, lifestyle and clinical characteristics of the studied group at baseline according to baseline PA level. Except for triglycerides (p for trend <0.05) and PA characteristics the three studied groups were comparable according to the analyzed variables at baseline. No statistical differences were found in the family history of CVD and the mean age of participants at final examination (mean age 59.7±9.0 years). Except for the highest active group (EE above 3840 kcal/week), most participants maintained their PA level throughout the observation. The mean EEs at final examination were 1302.8±775.4, 2705.3±1712.1 and 4384.7±2897.4 kcal/week in the low-to-moderate, high and very high PA groups, respectively (p<0.001).

**Table 1:** Distribution of demographic, lifestyle and clinical characteristics of the studied group at baseline (1985/90) according to physical activity level.

	Physical activity at baseline (tertiles of exercise-related energy expenditure)		
	low-to-moderate	high	very high
	(<2050 kcal/week)	(2050-3840 kcal/week)	(>3840 kcal/week)
	n = 33	n = 34	n = 34
Age, years	36.1±7.4	35.3±6.8	34.9±6.3
Current smokers, n	8	6	3
BMI, kg/m^2^	25.1±3.1	24.9±2.9	23.8±2.1
WC, cm	86.0±6.7	85.3±6.9	84.9±7.7
% fat	17.1±4.2	16.9±3.9	15.3±2.6
SBP, mmHg	122.3±11.9	118.2±14.7	120.2±15.6
DBP, mmHg	80.1±6.4	77.2±5.6	76.9±8.8
TC, mmol/l	4.91±0.51	4.83±0.57	4.84±0.58
LDL, mmol/l	3.11±0.55	2.92±0.54	2.83±0.55
TG mmol/l	1.45±0.59	1.22±0.52	1.13±0.37*
HDL mmol/l	1.26±0.17	1.37±0.33	1.34±1.13
Glucose, mg/dl	4.51±5.72	4.45±0.54	4.58±0.55
TC/HDL-C ratio	3.8±1.5	3.6±1.9	3.6±1.3
Physical activity (hours/week)			
Low/moderate intensity (≤6 METs)	3.1±2.8	2.7±3.1	2.7±3.9
High/very high intensity (>6 METs)	0.8±0.9	5.2±2.2	8.3±3.2***
Exercise-related energy expenditure (kcal/week)	1018.3±661.8	2813.0±499.3	6100.0±2807.8***
VO_2_max (ml/kg/min)	37.7±5.6	40.8±8.9	43.8±9.1*

Data presented as mean ± SD unless otherwise stated;

*p<0.05;

**p<0.01;

***p<0.001.

Abbreviations: BMI  =  body mass index; WC  =  waist circumference; SBP  =  systolic blood pressure, DBP  =  diastolic blood pressure, TC  =  total cholesterol.

There were significant differences in traditional CVD risk factors and subclinical atherosclerosis indices according to PA patterns during the observation (Table 2). During the 25-year observation most of the selected CVD risk factors worsened, mainly in the group of men with the lowest PA level. However, the number of regular smokers fell and HDL-C level increased substantially in all groups between the baseline and final examination. When we analyzed the groups with stable (maintained) PA level throughout the observation, a significant inverse correlation was found for the comparison between PA level and mean values of WC, % of fat tissue and lipids (Table 2). Generally, the most favorable CVD profile was found among men with maintained high PA (EE 2050–3840 kcal/week) with no case of obesity, metabolic syndrome or diabetes observed at final examination. Hypertension and hypercholesterolemia were found among 13.2% and 61.1% in the high PA group. Prevalence of hypertension, hypercholesterolemia, obesity and metabolic syndrome was 27.6%, 66.7%, 13.3% and 17.2% among men with low-to moderate PA, and 29.4%, 55.2%, 5.9% and 3.0% among men with very high PA.

**Table 2:** Traditional CVD risk factors and indices of subclinical atherosclerosis during final examination (2011/12) according to physical activity patterns during 25-year observation.

	PA level at baseline						
	low-to-moderate		high			very high	
Variable	(<2050 kcal/week)		(2050–3840 kcal/week)			(>3840 kcal/week)	
	maintained	increased	decreased	maintained	increase	decreased	maintained
	n = 26	n = 7	n = 5	n = 21	n = 8	n = 19	n = 15
Age, yrs	61.3±9.0	62.7±8.6	59.3±9.2	58.8±8.8	60.0±8.6	59.7±9.6	58.8±8.1
Current smokers, n	1	0	1	0	0	1	2
BMI, kg/m^2^	26.9±2.7	25.7±2.4	26.9±3.3	25.0±3.2	24.9±6.2	24.4±3.3	24.2±3.0
WC, cm	98.8±8.3	94.7±6.2	97.9±9.5	93.0±9.1	92.9±8.9	89.0±8.8	88.3±7.7**
% fat	21.9±4.2	19.0±5.2	22.9±5.9	19.2±5.1	18.1±6.3	17.9±5.1	17.5±5.6*
SBP, mmHg	132.2±14.7	129.2±16.1	130.0±10.3	124.0±11.1	124.0±11.1	126.4±15.1	125.4±14.9
DBP, mmHg	82.2±6.2	80.9±6.8	83.7±7.2	79.7±6.1	78.7±7.5	78.9±8.3	76.8±7.3
TC (mmol/l)	5.65±1.08	5.49±1.07	5.66±1.09	5.48±0.85	5.56±1.16	5.44±0.82	5.48±0.87
LDL(mmol/l)	3.61±1.06	3.53±1.00	3.58±0.71	3.37±0.71	3.32±0.76	3.16±0.66	3.28±0.69*
TG (mmol/l)	1.32±0.49	1.28±0.38	1.27±0.56	1.16±0.57	1.13±0.59	1.03±0.46	1.02±0.41**
HDL(mmol/l)	1.41±0.39	1.46±0.33	1.41±0.37	1.36±0.40	1.71±0.45^a^	1.76±0.48	1.81±0.45**
Glucose (mmol/l)	5.25±1.08	5.02±1.11	5.22±0.43	4.84±0.48	4.74±0.38^a^	4.78±0.38	4.73±0.33**
CAC, Agatston units	286.1±361.9	103.9±166.5	101.8±123.0	10.7±28.9	102.1±255.4	58.5±139.7	106.1±278.3
(median)	(121.3)	(20.4)	(48.9)	(1.7)***	(0.0)	(2.1)	(6.30)*
0, n	1	3	1	10**	5	10	6
1–100, n	13	3	1	10	2	8	5
>100, n	12	1	3	1**	1	1*	4*
IMT, mm	0.751±0.19	0.713±0.25	0.700±0.12	0.641±0.26	0.662±0.34	0.633±0.28	0.750±0.60
> 0.9, n	5	2	2	0	2	1	1
RHI	1.69±0.4	1.86±0.2	2.02±0.5	2.00±0.4	2.14±0.4	2.04±0.6	2.13±0.5
<1.67, n	12	1	1	0**	1	2*	3

Data presented as mean ± SD unless otherwise stated;

^a^ p<0.05 for comparisons within PA categories;

*p<0.05;

**p<0.01;

***p<0.001 for comparisons between the groups with stable (maintained) PA throughout the observation; Abbreviations: CAC  =  coronary artery calcification; IMT  =  intima-media thickness; RHI  =  reactive hyperemia index; other abbreviations as in Table 1.

Coronary artery calcification was significantly related to IMT (r = 0,502; p<0,001), while no substantial relationship was observed between CAC and RHI in the whole studied cohort.

The three analyzed measures of subclinical atherosclerosis were significantly related to several PA characteristics. However we have not found a linear relationship between EE and CAC, IMT or RHI.

Mean values of CAC in the groups with maintained low-to-moderate, high and very high PA were 286.1±361.9, 10.7±28.9, and 106.1±278.3, respectively (p<0.001 low-to-moderate vs high; and p<0.05 low-to-moderate vs very high). Almost half of men with high and very high PA, whereas only 12,1% of men with low-to-moderate PA level had a CAC score  =  0. Significant coronary calcification (CAC>100) was twice more often in the lowest activity group as compared to other participants. No substantial differences were found in the comparisons between the group according to the mean IMT. Men with maintained high PA had lower (but not significant) mean IMT (0.641±0.26 mm) than moderately or very active subjects. No important differences were noticed in the IMT between the low-to-moderate and very high PA groups (0.751±0.19 and 0.750±0.60 mm, respectively). An inverse trend in the relationship between EE and RHI was found in the whole study group (p for trend = 0.0501). Mean values of RHI in the groups with maintained low-to-moderate, high and very high PA were 1.69±0.4, 2.00±0.4, and 2.13±0.5, respectively.

Generally, the most favorable profile was observed among men who maintained their high PA during the observation (2050–3840 kcal/week). High proportion of a negative CAC as well as no case of CAC>400, IMT>0.9 and RHI<1.67 was observed in this group. Significant coronary calcification (CAC>400) was observed in men with the lowest and highest PA, i.e. in 24.0% of men who maintained low-to moderate PA (mean EE, 743±312 kcal/week) and in 20.0% of men who maintained very high PA (mean EE, 6105.0±2307.5 kcal/week).

Tables 3 and 4 show average PA during different life periods assessed by the historical PA questionnaire and correlation coefficients of traditional CVD risk factors and indices of subclinical atherosclerosis to PA during different life periods. Average number of hours per year spent on PA during younger periods of life (18–34 and 35–49 years of age) was comparable, while there was a significant increase in PA level at the age ≥50 years (p<0.01). Anthropometric measures, TG, TC/HDL-C, SBP, uric acid were negatively, whereas HDL-C and VO_2max_ were positively correlated with several historical PA indices. CAC and RHI were related to all measures of historical PA questionnaire, while IMT only to those historical measures which pertained to adolescence and young adulthood.

**Table 3:** Correlation coefficients of traditional CVD risk factors to LTPA in different life periods assessed by historical PA questionnaire.

	Life periods					
	12-34^th^ year of life	35-49^th^ year of life	≥50^th^ year of life	≥12^th^ year of life	Last 5 years	Last10 years
PA (hours/yr), mean±SD	209.6±168.9	234.3±156.7	253.2±149.9	215.1±122.6	255.5±150.7	257.6±149.8
BMI	−0.26**	−0.36***	−0.28**	−0.27**	−0.18	−0.21*
WC	−0.30**	−0.39***	−0.37***	−0.31**	−0.24*	−0.28**
% of body fat	−0.19*	−0.34***	−0.26*	−0.34**	−0.19*	−0,22*
HDL-C	0.21*	0.27**	0.23*	0.29**	0.23*	0.20*
TC/HDL-C	−0.21*	−0.26**	−0.29**	−0.31**	−0.29**	−0.25*
TG	−0.19	−0.27**	−0.28**	−0,33**	−0.23*	−0.24*
Glucose	−0.26**	−0.15	−0.13	−0.17	−0.12	−0.06
SBP	−0.18	−0.21*	−0.22*	−0.21*	−0.21*	−0.18
DBP	−0.17	−0.22*	−0.19	−0.16	−0.13	−0.14
Uric acid	−0.08	−0.22*	−0.25*	−0.18	−0.22*	−0.18
Vo2 max	0.23*	0.37**	0.39**	0.34**	0.33**	0.37**

Abbreviations as in Table 1.

*p<0.05;

**p<0.01;

***p<0.001.

**Table 4:** Correlation coefficients of subclinical atherosclerosis indices to LTPA in different life periods assessed by historical PA questionnaire.

	Atherosclerosis indices		
	CAC	IMT	RHI
12-34^th^ year of life (hours/year)	−0.25**	−0.21*	0.34***
35-49^th^ year of life (hours/year)	−0.24*	−0.13	0.37***
≥50^th^ year of life (hours/year)	−0.27**	−0.08	0.34***
≥12^th^ year of life (hours/year)	−0.26**	−0.16	0.29**
Last 5 years (hours/year)	−0.29**	−0.10	0.29**
Last 10 years (hours/year)	−0.27**	−0.16	0.28**

*p<0.05;

**p<0.01;

***p<0.001.

## Discussion

This is the first research investigating the influence of habitual PA on subclinical atherosclerosis in 25-year observation using a comprehensive panel of diagnostic procedures. Apart from regular assessment of current PA, we used a well-validated questionnaire for historical PA together with three recognized measures of subclinical atherosclerosis, and a set of the most important risk factors for cardiovascular and metabolic diseases.

The major finding of this study is that regular stable high PA with energy expenditure above 2050 kcal per week may counteract the age-related deterioration of traditional CVD risk factors as well as a development of atherosclerosis in men. However, the relationship between PA and subclinical atherosclerosis indices was nonlinear. We found that the most favorable profile was associated with the intermediate PA level, i.e. with EE not exceeding 3840 kcal/wk (mean EE of 2919.3±651.3 kcal/wk). Higher PA levels have not resulted in further substantial improvement of the obtained results. Another important observation is that PA affects the indices of subclinical atherosclerosis to a different extent. There was a strong correlation between lifetime PA, CAC and RHI, while statistical significance for IMT was relatively weak (p = 0.048) and contributed only to the period of PA in adolescence. Influence of weekly energy expenditure was also clearer in relation to CAC and RHI than to measures of IMT.

Studies which have examined the relationship between PA level and subclinical atherosclerosis have typically adopted a cross-sectional design (or relatively short periods of observation), so direct comparison of our findings with other authors' results is difficult. However, significant correlation between PA and CAC obtained in our study is to some extent in line with the findings provided by Desai et al. (2004)[9]. In a volunteer sample of 520 asymptomatic middle-aged men with at least 2 metabolic risk factors, those who were involved in regular long-duration PA had a lower prevalence of CAC than sedentary or less active individuals [9]. In our study cohort we have not found clear linear relationship between PA and CAC. Subjects with low-to moderate PA had generally higher mean CAC and prevalence of advanced calcification than more active individuals, but the most favorable profile was observed in the group with stable intermediate PA level (2050–3840 kcal/week). Further increase in EE did not provide additional improvement in CAC. In the available literature we have not found studies assessing the influence of regular very high PA on the indices of subclinical atherosclerosis. Wilund et al (2008) reported no significant relationship between CAC and PA level in a cross-sectional analysis of 7 highly active, endurance-trained “master athletes” [13]. The study was extremely underpowered due to the limited sample size and only a modest inverse correlation was found between VO_2_max and the number of calcified lesions, and a trend for a correlation between VO_2_max and logCAC score. Interesting results were obtained by Sung et al. (2012) in a group of 8565 apparently healthy middle-aged men [26]. They found significantly higher prevalence of advanced CAC among those with greater habitual exercise frequency (exercising ≥3/week), although those with a frequent exercise habits had a significantly higher VO_2_max which is to some extent in line with our observations. The authors explained that this counterintuitive finding was probably a result of a healthier lifestyle chosen by individuals with higher CVD risk (for example patients with hypertension and diabetes exercised more frequently than those without). In our study, men with a stable very high PA (EE>3840 kcal/week, exercising above 10 hours/week) had also significantly higher VO_2_max than less active subjects, although their mean CAC and prevalence of advanced CAC was less favorable than among those with intermediate PA. However, we cannot find an explanation of this unexpected finding as both groups with high and very high PA were comparable in CVD risk profiles. Further studies in larges sample of highly active men are needed to address our results.

Contrary to our results, a few other authors reported no association between PA and CAC [11], [12]. Bertoni et al. (2008) found no association between any domain or intensity of PA and CAC among 6814 participants of the Multi-Ethnic Study of Atherosclerosis (MESA)[27]. Only a walking pace remained favorably related to CAC after adjustment for potential confounders. On the other hand, Hamer et al. (2010) observed significant inverse association between objectively measured (but not self-reported) walking pace and CAC suggesting that short-distance walking speed may influence subclinical atherosclerosis [28].

Unexpectedly, carotid IMT was not substantially associated with PA levels in our study cohort. Although individuals with stable high PA had lower IMT than other participants of the study, the correlation did not reach the level of significance (p = 0.0502). However, it should be noted that even small difference in IMT may have substantial impact on incidence of CVD events. According to a meta-analysis performed by Lorenz et al. (2007) even a 0.10-mm carotid IMT difference was associated with an increased risk of stroke and myocardial infarction [8]. Importantly, no case of carotid atherosclerosis was found in the cohort with stable high associated with EE 2050–3840 kcal/week. The only statistically significant correlation was noticed between PA level during adolescence (according to the historical questionnaire) and the mean IMT assessed at the middle-age.

Although most other authors indicated an inverse correlation between leisure-time and sports activity and presence or progression of carotid atherosclerosis [10], [29], [30] there are also studies reporting no or modest associations [11], [27], [31], [32]. Bertoni et al. (2008) noticed significant relationship only between walking pace and IMT among middle-aged and older participants of MESA while there were no correlations across different domains and intensity of PA [27]. In the Atherosclerosis Risk in Communities Study an inverse association was found between carotid IMT and occupational (but not leisure-time and sports) PA in subjects aged 45–64 at baseline [11]. Neither intensity nor duration of PA were associated with IMT in older adults participating in the Cardiovascular Health Study [32]. Inconsistent results were also found in the results of studies examining the effect of exercise training on carotid IMT. The analysis Tanaka et al. (2002) and most subsequent studies (including longitudinal observations) found no difference in IMT between endurance-trained subjects and sedentary cohorts and indicated no evidence that exercise training altered arterial wall thickness in middle-aged and older men [33]–[35]. However, a six-year randomized, controlled trial of 140 middle-aged men (involved in aerobic exercise training or no intervention) showed about 40% decrease in progression of carotid IMT after excluding patients taking statins [36].

We are not aware of any study assessing the relationship between PA level and endothelial function using a peripheral arterial tonometry device. Hamburg et al. (2008) performed a cross-sectional analysis between traditional CVD risk factors and digital vascular function, but they have not included PA level into their models [37]. According to several previous reports using other methodology, PA level had an important influence on endothelial function and might counteract the loss of vasodilatory function associated with aging [38], [39].The beneficial effect of physical activity is suggested to be mediated by exercise-induced enhancement of blood flow, leading to augmented shear stress. As a consequence nitric oxide production and bioavailability are stimulated which results in arterial structural adaptations [40]. In response to exercise, the endothelium may release substances that modulate vascular tone, structure or blood characteristics and therefore influence CVD profile. However, the amount and intensity of physical activity required to improve endothelial function is unknown. Among various methods of evaluating endothelial function, non-invasive measurement of peripheral vasodilator response with fingertip PAT technology (EndoPAT) is thought to be a useful method for assessing vascular function in various populations [23], [24], [37].

Our results confirm previous reports indicating a beneficial influence of exercise on endothelial function. In the whole study cohort combined, there was a strong relationship between RHI and PA level in all assessed periods of life according to the historical questionnaire. Although no significant associations were noticed between the PA subgroups, there was a trend suggesting an inverse relationship between EE and RHI. Low-to moderate PA associated with EE below 2050 kcal/week seems to be insufficient in preventing endothelial dysfunction as almost half of this group had RHI<1.67 while no such case was found in subjects with stable high PA. Interestingly, we noticed endothelial dysfunction among about one third of the most active men with EE above 3840 kcal/week throughout the observation.

We have not found significant relationship between CAC and RHI in our cohort which is, to some extent, in line with the observation of Han et al. (2010) who concluded that these 2 markers may represent separate, independent processes in the progression of coronary atherosclerosis [41]. However, no case of endothelial dysfunction was found only among men with the best CAC profile i.e. men representing stable high PA.

According to our previous reports maintaining favorable cardiometabolic profile is associated with at least high habitual PA level. Although we observed health benefits related to lower PA levels (1000–2000 kcal/week) as compared to sedentary lifestyle, substantial prevention of CVD was achieved with EE above 2000 kcal/week [15], [42]. In the present study, vast majority of the whole cohort meet current recommendations for PA as only 15 individuals could be classified as sedentary or low active (regular EE<1000 kcal/week). However, regular EE below 2050 kcal/week occurred not sufficient to maintain as beneficial cardiometabolic profile as men representing higher PA level. An apparently unexpected finding was that the most active men (with EE above 3840 kcal/week) did not differ significantly in cardiometabolic risk from the men with intermediate PA. Importantly, there is a rising evidence that intensity rather than exercise frequency substantially influence health outcomes. Several papers analyzing the results of the Harvard Alumni Health Study suggested that light activities (<4 METs) were not associated with reduced CVD risk or mortality rates, moderate activities (4-<6 METs) appeared somewhat beneficial, and vigorous activities (≥6 METs) clearly predicted better health indicators and lower mortality rates [43], [44]. Further reports of other authors indicated if total EE of exercise was held constant, exercise performed at a vigorous intensity appeared to convey greater cardioprotective and metabolic benefits than exercise of a moderate intensity [45]–[47]. In our groups with stable high and very high PA level, both cohorts were involved mainly in vigorous exercises which could partially explain the obtained results.

Although most studies examining the influence of PA on CVD risk confirm that “some physical activity is better than none” and “additional benefits occur with more physical activity”, little is known about the impact of regular very high PA level substantially exceeding the guidelines. Current international recommendations for PA and exercise in adults suggest that increasing a minimum dose of PA (i.e. 30–45 minutes of moderate-intensity exercise on 5 days weekly or 20 minutes of vigorous-intensity exercises on 3 days weekly) may provide additional health benefits [4]. However, no “safe” threshold of PA has ever been distinguished. Sattelmair et al. (2011) indicated that men who were physically active at five times the basic guideline had only minimally lower risk of coronary heart disease than moderately active subjects suggesting relatively modest additional benefits of increasing PA level [48]. In our cohort with stable very high PA exercise-related EE was several times higher than current advanced guidelines. We have not found additional benefits of such high PA, and even observed a trend of worsening of some indices of subclinical atherosclerosis. Due to a rising interest in participation in regular vigorous trainings as well as long-lasting endurance events (including marathons and ultramarathons), further studied should be also focused on health impact of very high PA.

Several shortcoming of the present study should be acknowledged. Well known limitation is related to self-reported questionnaires on PA which are prone to recall bias. Subjects may overestimate recent PA and either forget to report or underestimate historical PA. This may explain lower PA reported in early as compared to advanced adulthood. As our study is not large-scale and randomized, the obtained results are not representative for middle-aged men. Participants of this study declared much higher level of PA in comparison to national statistics [49]. Due to a sample size, statistical power was limited for several analysis in selected subgroups.

The distinct strength of the study is the long follow-up period. Precise selection of the study participants as well as comprehensive assessment of healthy behaviors (including estimation of metabolic cost of PA) and clinical characteristics during the observation are also among important advantages. We excluded individuals taking drugs modifying CVD risk which additionally reduced the confounding effect of anti-atherogenic treatment. As atherosclerosis evolution is prolonged and occult, we decided to use a variety of tools assessing preclinical disorders even at the earliest stages. Comparable initial CVD risk in the studied groups additionally reduced the confounding effect of subclinical diseases at baseline. Longitudinal observation of a homogenous group according to ethnicity, socioeconomic status, work-related PA, dietary patterns and alcohol consumption enable to eliminate the risk associated with such known confounders as age, social class, lifestyle choices etc.

In summary, our study demonstrate that lifetime high PA may counteract the age-related development of atherosclerosis among healthy middle-aged men. Energy expenditure below 2050 kcal/week was not sufficient in preventing subclinical disorders. The most favorable results of atherosclerosis indices, as measured by CAC, IMT and RHI, were associated with EE 2050–3840 kcal/week. Further increase in PA level does not provide additional benefits. Although physical inactivity remains among the major problems worldwide, there is a need to investigate the influence of very high PA on indices of atherosclerosis.
